# Loneliness, Social Networks, and Health: A Cross-Sectional Study in Three Countries

**DOI:** 10.1371/journal.pone.0145264

**Published:** 2016-01-13

**Authors:** Laura Alejandra Rico-Uribe, Francisco Félix Caballero, Beatriz Olaya, Beata Tobiasz-Adamczyk, Seppo Koskinen, Matilde Leonardi, Josep Maria Haro, Somnath Chatterji, José Luis Ayuso-Mateos, Marta Miret

**Affiliations:** 1 Department of Psychiatry, Universidad Autónoma de Madrid, Madrid, Spain; 2 Instituto de Salud Carlos III, Centro de Investigación Biomédica en Red de Salud Mental, CIBERSAM, Madrid, Spain; 3 Department of Psychiatry, Hospital Universitario de La Princesa, Instituto de Investigación Sanitaria Princesa (IP), Madrid, Spain; 4 Parc Sanitari Sant Joan de Déu, Barcelona, Spain; 5 Department of Medical Sociology, Jagiellonian University Medical College, Krakow, Poland; 6 National Institute for Health and Welfare, Helsinki, Finland; 7 Fondazione IRCCS, Neurological Institute Carlo Besta, Milano, Italy; 8 Universitat de Barcelona, Barcelona, Spain; 9 Department of Health Statistics and Information Systems, World Health Organization, Geneva, Switzerland; National Institute of Child Health and Human Development, UNITED STATES

## Abstract

**Objective:**

It is widely recognized that social networks and loneliness have effects on health. The present study assesses the differential association that the components of the social network and the subjective perception of loneliness have with health, and analyzes whether this association is different across different countries.

**Methods:**

A total of 10 800 adults were interviewed in Finland, Poland and Spain. Loneliness was assessed by means of the 3-item UCLA Loneliness Scale. Individuals’ social networks were measured by asking about the number of members in the network, how often they had contacts with these members, and whether they had a close relationship. The differential association of loneliness and the components of the social network with health was assessed by means of hierarchical linear regression models, controlling for relevant covariates.

**Results:**

In all three countries, loneliness was the variable most strongly correlated with health after controlling for depression, age, and other covariates. Loneliness contributed more strongly to health than any component of the social network. The relationship between loneliness and health was stronger in Finland (|β| = 0.25) than in Poland (|β| = 0.16) and Spain (|β| = 0.18). Frequency of contact was the only component of the social network that was moderately correlated with health.

**Conclusions:**

Loneliness has a stronger association with health than the components of the social network. This association is similar in three different European countries with different socio-economic and health characteristics and welfare systems. The importance of evaluating and screening feelings of loneliness in individuals with health problems should be taken into account. Further studies are needed in order to be able to confirm the associations found in the present study and infer causality.

## Introduction

The association between social relationships and health is well documented and has been of interest to the scientific community for many years [1–3]. Much of the earlier literature used different concepts interchangeably, such as feeling lonely, living in a single household, having few social contacts or a small social network, or not having people to trust; however, recent studies have made important advances by moving beyond simple indicators related to marital status or living arrangements, to analyze different dimensions and dynamics of social networks [4, 5] and separating these effects from those of feelings of loneliness [6].

There is considerable evidence that the nature and extent of an individual’s social network, such as quantity and quality of social relationships [7, 8] and frequency of contact [9], can have a significant impact on health. An extensive social network has been shown to be a protective factor against dementia [2, 4]. Furthermore, older people who are married or cohabiting and those with high levels of trust and solidarity, as well as those with medium-to-high psychological resources, all experience better self-rated health [10]. Social networks and social support are related, since they are part of the same construct [11]; however, they focus on different aspects and should be evaluated separately. Litwin and Landau [12] found that the significance of the social network predicts the availability of social support. A systematic review carried out by Santini, et al. [11] investigated the association between social relationships and depression, and found that social networks play a protective role against depression, just as social support does.

On the other hand, loneliness may have deleterious effects on health [13, 14]. Lonely individuals have lower cardiovascular contractility, heart rate, and cardiac output than non-lonely individuals [13]; they are also more likely to present alterations in the immunological system [15] and obesity [16]. Loneliness is also associated with poorer sleep efficiency and quality [13, 15], depressive symptomatology [14], alcoholism [17], Alzheimer’s disease [18], and suicidal ideation and behavior [19]. Furthermore, some studies report that lonely individuals also show an increased risk of all-cause mortality [20, 21].

Although previous evidence shows that social networks and loneliness have effects on health [22], there is still a need to know whether the relationship between the subjective perception of loneliness and health is different from the relationship between each component of the social network and health, after controlling for potential confounders, and to analyze with identical methods whether these relationships are different across countries with different population, health, and socio-economic characteristics and family structures. International studies have clearly documented the difference in health across countries with different social welfare systems [23]. Earlier studies analyzing loneliness [24, 25] and social networks [26] found differences across countries. Moreover, differences across generations have been documented in previous studies. Jylha [27] found that age is related to negative life changes that increase loneliness and weaken social integration, whereas Carstensen [28] suggested that although social networks grow smaller with advancing age, they also grow more satisfying.

In addition, more research is needed to better understand the differences between the concepts by analyzing separately loneliness and the number of contacts with members of the network, since these are two different concepts: loneliness is a subjective feeling, and the number of contacts is an objective aspect [6, 29]. Previous studies found that the subjective experience of loneliness is more harmful to health than the actual number of the social contacts that a person has [29]. A longitudinal study found that loneliness predicts changes in depressive symptoms, and the association between these variables is not attributable to objective social isolation, emotional closeness in relationships or social support [14]. Although social networks have been well documented and loneliness is now being increasingly studied, to our knowledge few studies have been carried out that analyze both variables at the same time (loneliness and size of the network), much less that disentangle and analyze separately the other components of the social network: frequency and quality of contact.

The present study aims to: a) disentangle the differential associations of health with the different components of the social network (size and quality of the network, and frequency of contact with members of the network) and the subjective perception of loneliness; b) analyze the additional explanatory power of each of the elements in their association with health status; and c) examine whether this association differs across countries.

The hypotheses postulated are: a) the components of the social network and the subjective perception of loneliness will be associated with health status; b) loneliness will be more associated with health than the size, frequency and quality of social networks; c) the association between the aforesaid variables and health status will be different across the countries considered in this study, due to their different social protection systems, economic situations, social network structures, and family ties.

## Method

### Design

The Collaborative Research on Ageing in Europe (COURAGE in Europe) project (http://courageproject.eu/) [30] is a European Union-funded, cross-sectional household survey of a probabilistic sample representative of the adult population aged 18+ years, in three European countries (Finland, Poland and Spain). Nationally representative samples were obtained for each of the three countries according to the procedure described below. These countries were selected to give a broad representation of different European regions, representing Northern, Eastern and Southern Europe according to the classification of the United Nations [31], and different demographic, cultural, socio-economic and health characteristics, as well as different social welfare systems [23].

### Sample and procedure

Participants were interviewed face-to-face in their own homes, with Computer-Assisted Personal Interviewing. The surveys were conducted between April 2011 and May 2012. The COURAGE survey questionnaire was translated from English into Finnish, Polish, and Spanish following the World Health Organization translation guidelines for assessment instruments. These include a forward translation, a targeted back-translation, review by a bilingual expert group, and a detailed translation report. The questionnaire used in the present study is shown in S1 Appendix. Quality assurance procedures were implemented during fieldwork [32].

A multistage clustered design was used to obtain nationally representative samples in each country. In Poland and Spain, a stratified multistage random sampling method was used and strata were created according to the geographical administrative regions and number of people living in the geographical area. Age strata were used to select households according to the age structure of the population. The respondents were randomly selected among inhabitants of a household from a certain age group. In Finland, the design was a stratified two-stage cluster sampling design, and strata were created based on the largest towns and university hospital regions. A systematic sampling of people was conducted so that the sample size in each stratum was proportional to the corresponding population base. Although the samples were representative of the population of the three countries, the group of people older than 80 years was overrepresented in the sampling in order to avoid having a small sample size of the oldest old.

A total of 10 800 individuals participated in the survey: 1976 from Finland, 4071 from Poland, and 4753 from Spain. The individual response rate was 53.4% for Finland, 66.5% for Poland, and 69.9% for Spain. The present study was approved by the ethical committee of Neurological Institute Carlo Besta, Milan, Italy, project coordinator; the Ethics Review Committee, National Public Health Institute, Helsinki, Finland; the Bioethical Committee, Jagiellonian University, Krakow, Poland; Ethics Review Committee, Parc Sanitari Sant Joan de Déu, Barcelona, Spain; and Ethics Review Committee, La Princesa University Hospital, Madrid, Spain. Written informed consent from each participant was also obtained.

### Measures

Loneliness was assessed by means of the 3-item UCLA Loneliness Scale [33]. This scale comprises the following items: *How often do you feel that you lack companionship?, How often do you feel left out?* and *How often do you feel isolated from others?*, which are assessed on a 3-point scale (1 = hardly ever; 2 = some of the time; 3 = often). The UCLA Loneliness Scale has shown satisfactory reliability and both concurrent and discriminant validity [33]. The scale showed acceptable internal reliability in the present study (Cronbach's alpha = 0.84; mean inter-item correlation = 0.65). The scores for each item were added up to produce a loneliness score ranging from 3 to 9, with higher scores indicating higher loneliness levels.

A detailed description of the individual’s social network was obtained. It included the following components: 1) size of the network; 2) frequency of contact with members of the network; and 3) quality of the network. The three components of the social network considered are based on the structural dimension of the Berkman-Syme Social Network Index [34], which measures the number of social ties, closeness with members of the network, and frequency of contact. The size of the network was assessed by asking the participant about the number of people in the network (“*Please state the number of people [in total] who are so close to you at the present time that you*: *can talk to them about personal affairs*, *can get help from them in everyday matters*, *and/or enjoy spending your leisure time with them [please consider family members*, *friends*, *colleagues*, *etc*.*]”*). The variables frequency of contact with members of the network (also known as intensity of the network) and quality of the network were assessed with an index ranging from 0 to 8, asking the person if they had had contacts with the members of the network at least once per month in the previous 12 months and whether they had a close relationship with them. One point was assigned for each of the eight types of the relationship: spouse or partner, parents, children, grandchildren, other relatives, co-workers, friends, and neighbors. This scoring method is based on the Social Network Index proposed by Cohen [35], which assesses participation in several types of relationships.

Health status was assessed with a multi-domain health state measurement that considers that health is more than the absence of disease or injury; it also takes into account the ability to carry out physical and mental actions, and tasks [36]. This measure was developed after the World Health Organization (WHO) argued, “functioning and functioning domains constitute the operationalization that best captures our intuitive notion of health” [37]. This health measure has been previously used in the 70 countries considered in the World Health Survey and in the WHO Study on Global AGEing and adult health, to compare the health of the population around the world. It is a set of self-reported health-related questions that were grouped into eight health domains: vision, mobility, self-care, cognition, interpersonal activities, pain and discomfort, sleep and energy, and affect [36]. For each question, responses were recorded on a 5-point scale ranging from no difficulty/problem to extreme difficulty/inability. An overall health score from these health-related questions was obtained using a Rasch partial credit model [38]. The overall health score ranged from 0 to 100, where 0 represents the worst health and 100 represents the best health.

The presence of a depressive episode during the previous 12 months was assessed with a set of questions based on the World Mental Health Survey version of the Composite International Diagnostic Interview [39]. Individuals were considered to have had a depressive episode if they had been diagnosed with depression and had been taking medication or receiving some other treatment (e.g. psychotherapy) during the previous 12 months, or if they reported the presence of the core symptoms of the condition during the previous 12 months, according to ICD-10 Diagnostic Criteria for Research [40].

Participants were also asked to provide socio-demographic information: age, gender, years of education, residential setting (rural, urban), household composition (living in a single household, a dual household, or a household with three or more people), and household income. Marital status was dichotomized as either married or in a partnership, or not married or in a partnership (including single, divorced, widowed, or not living with a partner), similarly to other studies that also analyzed loneliness [41, 42]. A 5-level ordinal variable for household income was obtained, representing the quintile of household income according to the country. This variable was then dichotomized, with belonging to the first or the second quintile of household income considered as the reference category.

### Statistical analysis

All data were weighted to account for the sampling design in each country and to generalize the study sample to the reference population. Normalized weights for each age group (18–49 and 50+ years) were used. Post-stratification corrections were made to the weights to adjust for the population distribution obtained from the national census from each country, and for non-response [43]. Rates and means were calculated using the direct method of age standardization to the European standard population [44]. Robust standard errors were estimated using the Taylor series linearization method [45] to adjust for the effects of weighting and clustering.

Analysis of variance (ANOVA) tests and χ^2^ tests were used to assess differences across countries in socio-demographic characteristics, components of the social network, and loneliness. The mean estimates on the components of the social network, loneliness, and health status were obtained separately for each age group (18–49 and 50+ years) in order to take into account the specific sampling weights considered for each group. Differences across countries were assessed for each age group and for the overall population. Cramer’s *V* (χ^2^ test), Cohen’s *f* (ANOVA) and Hedges’ *g* (pairwise comparisons) were reported as effect size measures in case of significant differences at the 95% confidence level. Cohen’s guidelines [46] were used as standard to evaluate the magnitude of the effect size. Cramer’s *V* values of 0.10, 0.30, and 0.50 constitute small, medium, and large effect sizes; whereas these values are 0.10, 0.25, and 0.40, respectively, for Cohen’s *f*. Hedges’ *g* values of .20, .50, and .80, constitute small, medium, and large effect sizes, respectively.

Pearson correlation coefficients assessed the relationships between the components of the social network among themselves, and with loneliness. In order to look at the independent effect of social networks (in terms of size of the network, frequency of contact with members of the network, and quality of the network) and loneliness on health status, a hierarchical linear regression model was conducted in each of the three countries considered in the present study. A first block comprising socio-demographic variables and the presence of a depressive episode was included to account for their effect. Depression was added as a potential confounder identified in the literature, since it has long been recognized that loneliness and depressive symptoms are strong correlates [14, 47–51]. Moreover, Cacioppo et al. [48] concluded that loneliness and depressive symptoms could act in a synergistic way to reduce health. Then, the three components of the social network and the loneliness score were introduced in two different blocks to assess their differential association with health. The increase in the proportion of variance explained in each block (increase in the adjusted *R*^2^) was tested at each step by means of the difference in the likelihood ratio chi-square for each model, which tested the null hypothesis that each additional set of independent variables contributed nothing beyond the set(s) of variables entered in the model(s) at earlier steps. The Ordinary Least Squares (OLS) estimator, which trades robustness for some improvement in efficiency [52] and has been shown to yield the best fit of data [53], was used for each model. Beta coefficients were reported, and can be interpreted as change in the outcome (in standard deviations) per standard deviation change in the predictors; they were used to assess which variables had the highest association with the outcome variable.

A multiple linear regression model was estimated over the pooled sample, including only the variables that were found significant in at least one of the previous models conducted for each country. Dummy variables for countries were included in this model; moreover, interaction terms between countries and the variables related with social networks and loneliness were added to account for country differences with regard to the association of social networks and loneliness with health. Interaction terms between age (considered as a continuous variable) and loneliness, and between gender and loneliness, were also included.

Finally, gender differences in loneliness scores were assessed in each country by means of unpaired *t*-tests, reporting Hedges’ *g* as effect size measures. Loneliness scores for different age groups were also analyzed by country.

Data analysis was performed incorporating the sample weights and using Stata version 11.0. Stata's survey command (svy), which fits statistical models for complex survey data, was used. Confidence intervals (CI) for hypothesis tests were constructed at the 95% confidence level.

## Results

The final sample comprised 10 047 participants and was obtained after excluding the participants who did not answer the questions about their social network or their perception of loneliness. Even though the excluded sample (*n* = 753) did not differ by gender (57.4% women in the final sample vs. 56.6% women in the excluded sample, *p* = 0.67) or percentage of people living in a rural setting (26.7% vs. 25.0%, *p* = 0.25), the percentage of people married or in a partnership (59.1% vs. 53.0%, *p* = 0.002, Cramer’s *V* = 0.03) and the mean age (58.35 ± 16.77 in the final sample vs. 70.00 ± 17.41 in the excluded sample, *p* < 0.001, Hedges' *g* = 0.69) were significantly different, albeit associated with very small effect sizes. Table 1 shows the socio-demographic characteristics of the final sample separately by each country. In general terms, while the differences found in socio-demographics across countries were significant, they were also associated with a small effect size.

**Table 1:** Socio-demographic characteristics of the final sample, by country.

	Finland	Poland	Spain	Effect size
**Number of participants (*n*)**	1821	3851	4375	-
**Gender (%)**				0.05
Female	56.95	60.17	55.09	
Male	43.05	39.83	44.91	
**Age, Mean (SD)**	58.21(16.03)	56.96(17.94)	59.63(15.89)	0.07
**Current marital status (%)**				0.05
Not married	36.96	44.12	39.73	
Married or in partnership	63.04	55.88	60.27	
**Residential setting (%)**				0.30
Rural	22.02	43.34	13.94	
Urban	77.98	56.66	86.06	
**Years of education, Mean (SD)**	12.35 (4.25)	11.73 (3.82)	10.94 (6.28)	0.10
**Household composition (%)**				0.10
Living in a single household	29.21	25.58	19.45	
Living in a dual household	47.39	37.86	38.22	
Living in a household with three or more people	23.39	36.56	42.33	

Effect size: Cramer’s *V* for χ^2^ tests (categorical variables) and Cohen’s *f* for ANOVA tests (quantitative variables). Effect size was reported for all the differences that were found to be significant at the 95% confidence level.

As shown in Table 2, the lowest score on loneliness was found in Finland. The size of the network was greater in Finland and Spain, whereas the quality of the network was better in Poland than in Finland and Spain. Significant differences were found in quality of the network, frequency of contact, and loneliness in the 18–49 age group, whereas in the 50+ age group significant differences were found in loneliness, size and quality of the network, and frequency of contact. In all cases, the significant differences found were associated with a small effect size. No significant differences in the overall sample (pooling both age groups) were found across countries regarding frequency of contact. In general terms, the health status score (also shown in Table 2) was higher in Finland and lower in Poland. The only pairwise comparison in health status associated with a high effect size was found for the older age group, in which the Finnish sample showed a better health status than the Polish sample.

**Table 2:** Mean estimates (95% CI) on the components of the social network, the UCLA Loneliness Scale and health status.

Variables				*F*	d. f.	*p*	Hedges' *g*		
	Finland	Poland	Spain				F-P ^a^	F-S ^b^	P-S ^c^
***18–49 years***									
Size of the network	9.01 (8.30, 9.72)	8.02 (7.13, 8.92)	9.05 (8.44, 9.67)	1.94	2, 2402	0.14	n.s.	n.s.	n.s.
Frequency of contact	5.21 (5.09, 5.33)	5.31 (5.19, 5.43)	5.13 (5.04, 5.21)	3.01	2, 2402	0.049	n.s.	n.s.	0.13
Quality of the network	5.41 (5.30, 5.52)	5.66 (5.56, 5.76)	5.28 (5.19, 5.36)	16.19	2, 2402	<0.001	0.18	n.s.	0.27
Loneliness	3.50 (3.40, 3.59)	3.70 (3.60, 3.79)	3.60 (3.51, 3.69)	4.03	2, 2402	0.018	0.18	n.s.	n.s.
Health status	74.81 (73.67, 75.96)	71.56 (70.57, 72.55)	75.37(74.54, 76.20)	17.92	2,2402	<0.001	0.28	n.s.	0.32
***50+ years***									
Size of the network	8.39 (7.91, 8.87)	6.83 (6.46, 7.20)	8.33 (8.01, 8.66)	21.25	2, 7641	<0.001	0.22	n.s.	0.20
Frequency of contact	5.05 (4.97, 5.12)	4.82 (4.74, 4.91)	5.14 (5.09, 5.19)	20.40	2, 7641	<0.001	0.14	n.s.	0.21
Quality of the network	5.49 (5.41, 5.56)	5.53 (5.45, 5.61)	5.33 (5.28, 5.38)	11.59	2, 7641	<0.001	n.s.	0.12	0.13
Loneliness	3.51 (3.45, 3.57)	3.79 (3.73, 3.85)	3.74 (3.68, 3.80)	23.14	2, 7641	<0.001	0.22	0.17	n.s.
Health status	69.82 (69.29, 70.35)	61.34 (60.84, 61.85)	66.16 (65.73, 66.60)	261.72	2,7641	<0.001	0.76	0.30	0.39
***Overall sample***									
Size of the network	8.74 (8.29, 9.19)	7.50 (6.97, 8.03)	8.74 (8.36, 9.11)	8.15	2, 10045	<0.001	0.18	n.s.	0.16
Frequency of contact	5.14 (5.06, 5.21)	5.10 (5.02, 5.18)	5.13 (5.08, 5.19)	0.33	2, 10045	0.25	n.s.	n.s	n.s.
Quality of the network	5.44 (5.37, 5.51)	5.60 (5.54, 5.67)	5.30 (5.25, 5.35)	24.25	2, 10045	<0.001	0.10	0.10	0.20
Loneliness	3.50 (3.44, 3.56)	3.74 (3.68, 3,80)	3.66 (3.60, 3.72)	14.88	2, 10045	<0.001	0.19	0.12	n.s.
Health status	72.63 (71.94, 73.31)	67.08 (66.48, 67.68)	71.34 (70.83, 71.84)	86.09	2, 10045	<0.001	0.46	0.10	0.25

Weighted and age-standardized data

^a^ Effect size associated with significant differences found in the pairwise comparison between Finland and Poland

^b^ Effect size associated with significant differences found in the pairwise comparison between Finland and Spain

^c^ Effect size associated with significant differences found in the pairwise comparison between Poland and Spain

n. s. = Significant differences were not found and effect size is not reported

The relationships between the components of the social network (size of the network, frequency of the contact, and quality of the network) and loneliness were assessed in unadjusted analyses by means of a correlation matrix (Table 3). The strongest relationship was found between quality of the network and frequency of contact with members of the network: *r* = 0.71 [95% CI = (0.70, 0.72)]. The correlation between loneliness and size of the network was low (*r* = -0.11), whereas the correlation coefficients between loneliness and quality of the network (*r* = -0.24), and between loneliness and frequency of contact (*r* = -0.25) were moderate.

**Table 3:** Correlation matrix (95% CI) among size of the network, frequency of contact with members of the network, quality of the network, and the score on the UCLA Loneliness Scale (*n* = 10 047).

	Size of the network	Frequency of contact	Quality of the network	Loneliness
**Size of the network**	1	-	-	-
**Frequency of contact**	0.14 (0.12, 0.16)	1	-	-
**Quality of the network**	0.14 (0.12, 0.16)	0.71 (0.70, 0.72)	1	-
**Loneliness**	-0.11 (-0.13, -0.09)	-0.25 (-0.27, -0.23)	-0.24 (-0.26, -0.22)	1

A hierarchical linear regression model was estimated in each country to assess the differential association of loneliness and the components of the social network with health. Similar results were found across countries (Table 4). A significant increase in the percentage of variance explained was observed when loneliness was added to the model, but not when the block corresponding to the components of the social network was added. The strongest relationships with health were found for age, depression, and loneliness. The association of age with health was different across countries (β = -0.24 in Finland, β = -0.32 in Spain, and β = -0.47 in Poland). In all cases, a higher age, the presence of depression and a higher score on loneliness were associated with a worse health status. In Finland, the effect size associated with the relationship between loneliness and health status was higher (β = -0.25) than in Spain and Poland (β = -0.18 and β = -0.16, respectively). Frequency of contact with members of the network was the only component of the social network having a significant association with health, and it was included in the final multiple linear regression model.

**Table 4:** Final hierarchical linear regression models predicting health status in each country; weighted data.

	Finland		Poland		Spain	
Variables	Coef. (s. e.)	β	Coef. (s. e.)	β	Coef. (s. e.)	β
Intercept	81.85 *** (2.30)		81.45 ***(1.82)		81.65 ***(1.58)	
**First block**						
Gender (Ref. Female)	1.60 ** (0.46)	0.08	1.91 *** (0.45)	0.08	2.49 *** (0.37)	0.10
Age	-0.14 *** (0.02)	-0.24	-0.32 *** (0.02)	-0.47	-0.24 *** (0.01)	-0.32
Married or in a partnership (Ref. Not married or in a partnership)	1.58 (0.88)	0.07	-0.36 (0.69)	-0.01	-0.58 (0.52)	-0.02
Years of education	0.22 ***(0.06)	0.09	0.33 *** (0.07)	0.10	0.33 *** (0.03)	0.17
Residential setting (Ref. Rural)	1.22 * (0.53)	0.05	-0.16 (0.45)	-0.01	1.02 * (0.49)	0.17
Household composition (Ref. Living in a single household)						
Living in a dual household	-2.77 ** (0.91)	-0.13	-1.23 (0.70)	-0.05	-1.62 (0.56)	-0.07
Living in a household with three or more **People**	-3.30 ** (1.09)	-0.13	-2.91 *** (0.75)	-0.12	-2.47 *** (0.62)	-0.10
Household income (Ref. 1^st^/2^nd^ quintile)	1.71 ** (0.57)	0.08	1.99 *** (0.49)	0.08	0.73 * (0.36)	0.03
Depression (Ref. No)	-8.71 *** (0.68)	-0.25	-7.79 *** (0.65)	-0.18	-8.32 *** (0.46)	-0.27
**Second block, Δ*R***^**2**^	Δ*R*^2^ = 0.003, n.s.		Δ*R*^2^ = 0.006, n.s.		Δ*R*^2^ = 0.004, n.s.	
Size of the network	-0.02 (0.03)	-0.01	-0.04 (0.03)	-0.02	-0.03 (0.02)	-0.03
Frequency of contact	0.34 (0.23)	0.05	0.60 ** (0.18)	0.08	0.26 (0.20)	0.03
Quality of the network	0.22 (0.23)	0.03	0.05 (0.19)	0.01	0.33 (0.19)	0.04
**Third block, Δ*R***^**2**^	Δ*R*^2^ = 0.052 ||		Δ*R*^2^ = 0.022 ||		Δ*R*^2^ = 0.020 ||	
Loneliness	-2.48 *** (0.21)	-0.25	-1.50 *** (0.15)	-0.16	-1.46 *** (0.12)	-0.18
**Adjusted *R***^**2**^ **of the final model**	*R*^2^ = 0.288		*R*^2^ = 0.400		*R*^2^ = 0.383	

* *p* < 0.05

** *p* < 0.01

*** *p* < 0.001.

|| Indicates significant increase of variance explained at a 99% confidence level.

n.s. Indicates non-significant increase of variance explained at a 95% confidence level.

In the final model shown in Table 5, a high effect associated with the relationship between loneliness and health was found after controlling for covariates. A marginally significant effect for frequency of contact with members of the network was also found. Frequency of contact was associated with better health status. Regarding the other covariates, age (β = -0.47), depression (β = -0.23), and years of education (β = 0.13) presented the strongest correlation with health: higher age and the presence of depression were associated with worse health status, whereas more years of education were associated with better health. Men had a better health status than women (β = 0.13) and living in Finland was associated with better health.

**Table 5:** Multiple linear regression model with health status as dependent variable; weighted data.

	Coef. (s.e.)	95% CI	*t*	β
Intercept	93.44 *** (2.04)	(89.44, 97.44)	45.78	-
Gender (Ref. Female)	3.14 *** (0.69)	(1.78, 4.49)	4.54	0.13
Age	-0.33 *** (0.02)	(-0.38, -0.29)	-14.69	-0.47
Years of education	0.32 *** (0.03)	(0.26, 0.37)	11.54	0.13
Residential setting (Ref. Rural)	0.53 (0.29)	(-0.03, 1.09)	1.84	0.02
Household composition (Ref. Living in a single household)				
Living in a dual household	-1.44 *** (0.30)	(-2.02, -0.85)	-4.79	-0.06
Living in a household with three or more people	-2.50 *** (0.35)	(-3.19, -1.82)	-7.18	-0.10
Household income (Ref. 1^st^/2^nd^ quintile)	1.29 *** (0.27)	(0.77, 1.82)	4.84	0.05
Depression (Ref. No)	-8.38 *** (0.33)	(-9.04, -7.73)	-25.11	-0.23
Country (Ref. Finland)				
Poland	-12.27 *** (1.56)	(-15.33, -9.21)	-7.87	-0.50
Spain	-5.67 *** (1.54)	(-8.70, -2.64)	-3.67	-0.23
Frequency of contact	0.30 (0.16)	(-0.01, 0.61)	1.89	0.04
Frequency of contact # Poland	0.48 * (0.20)	(0.08, 0.87)	2.37	0.10
Frequency of contact # Spain	0.10 (0.21)	(-0.32, 0.49)	0.48	0.02
Loneliness	-3.72 *** (0.39)	(-4.49, -2.96)	-9.51	-0.41
Loneliness # Poland	1.05 *** (0.25)	(0.56, 1.53)	4.21	0.18
Loneliness # Spain	1.09 *** (0.23)	(0.64, 1.53)	4.79	0.19
Loneliness # Age	0.02 *** (0.01)	(0.01, 0.03)	4.15	0.19
Loneliness # Male	-0.31 (0.17)	(-0.64, 0.02)	-1.84	-0.05

* *p* < 0.05

** *p* < 0.01

*** *p* < 0.001

Interaction terms between frequency of contact with members of the network and country, between loneliness and country, between loneliness and age, and between loneliness and gender, were considered. Adjusted *R*^2^ of the model = 0.395.

**Fig 1:**
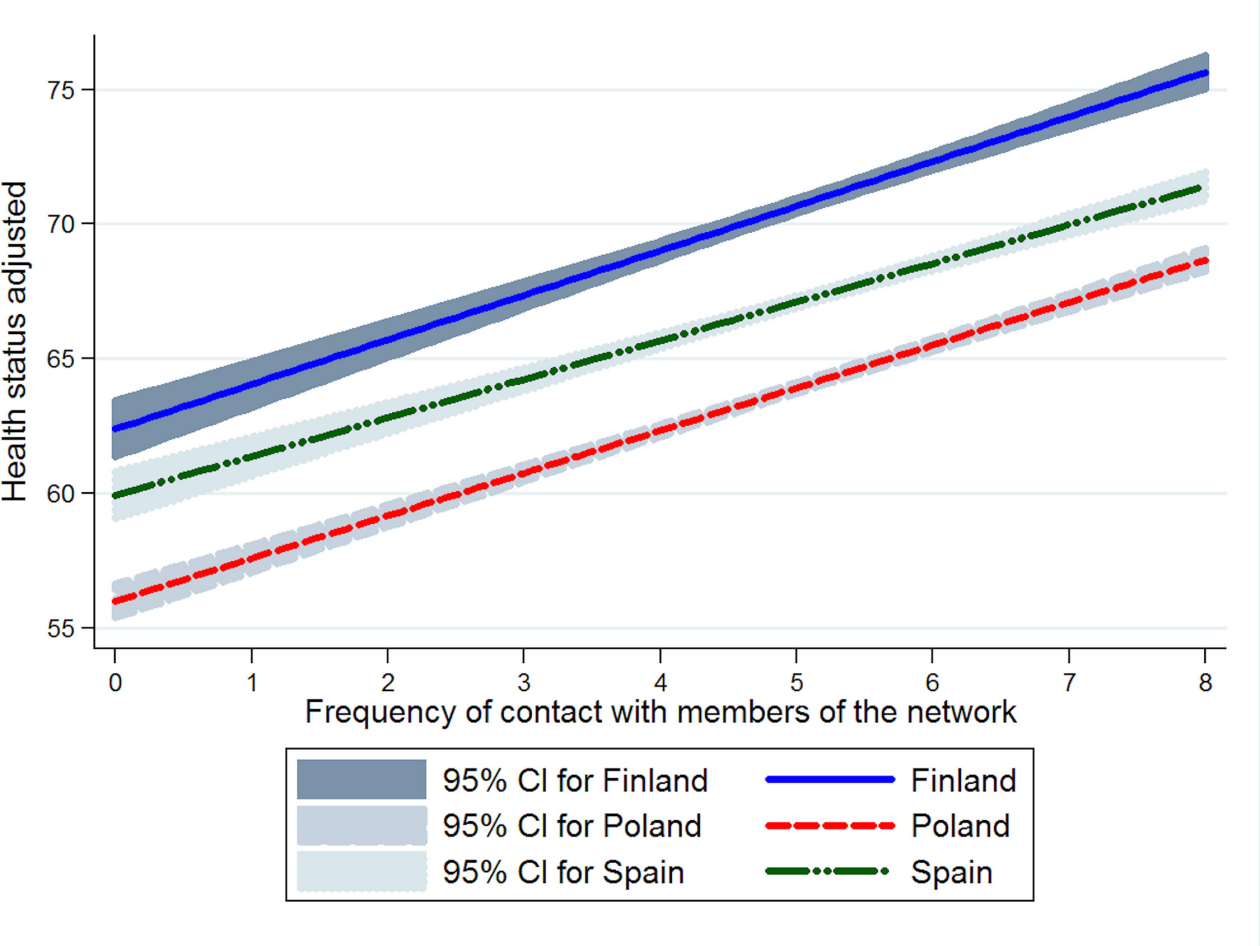
Relationship between frequency of contact with members of the network and health status by country, adjusted for the covariates considered in the multiple linear regression model.

**Fig 2:**
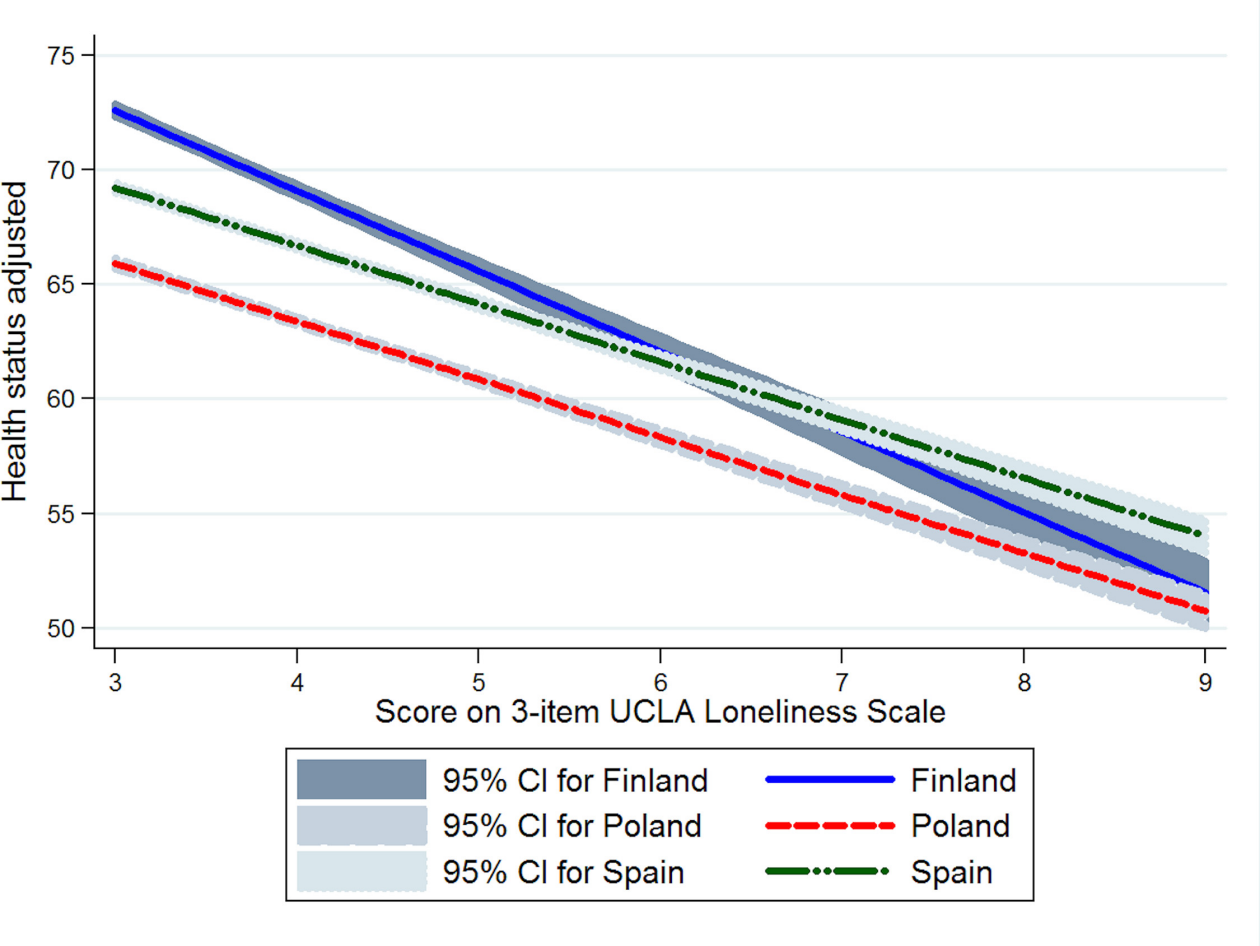
Relationship between loneliness and health status by country, adjusted for the covariates considered in the multiple linear regression model.

According to the interaction terms included in the last regression model, the relationship between frequency of contact with members of the network and health was slightly stronger in Poland than in Finland and Spain (Fig 1). These differences across countries were higher in the case of the relationship between loneliness and health: the association of loneliness with health was stronger in Finland than in the other two countries (Fig 2).

**Fig 3:**
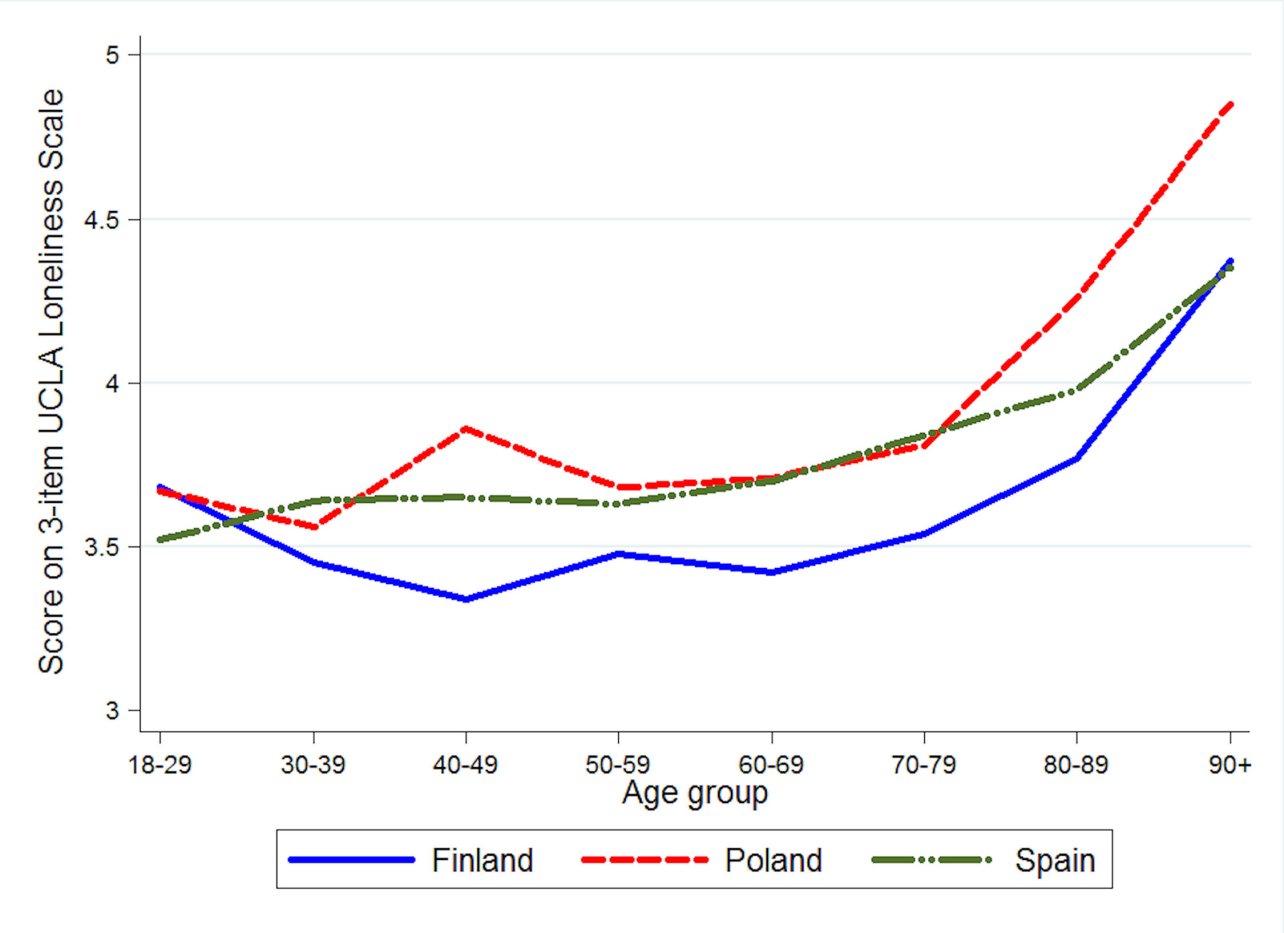
Mean score on 3-item UCLA Loneliness Scale, by country and age group.

As age increased, the relationship between loneliness and health was less strong, while the interaction term between gender and loneliness was not significant, as can be observed in Table 5. In all countries, loneliness mean scores were significantly higher in females than in males, although these differences had small effect sizes: in Finland, 3.55 ± 1.09 for females vs. 3.41 ± 0.94 for males (*p* = 0.003; Hedges' *g* = 0.14); in Poland, 3.91 ± 1.35 vs. 3.78 ± 1.28 (*p* = 0.003; Hedges' *g* = 0.10); and in Spain, 3.89 ± 1.59 vs. 3.56 ± 1.26 (*p* < 0.001; Hedges' *g* = 0.23). Loneliness mean scores by country and age group are shown in Fig 3. Eight different age groups were considered (18–29, 30–39, 40–49, 50–59, 60–69, 70–79, 80–89, and 90+) and a rising trend could be observed in the oldest population. Beta coefficients associated with the relationship between age group and loneliness were 0.04 in Finland, 0.11 in Poland, and 0.07 in Spain.

## Discussion

The results of the present study showed that there was a small or no difference across countries in the mean scores for loneliness and the components of the social network. People aged 50+ from Finland reported slightly lower scores for loneliness than their Polish and Spanish counterparts. This finding is in the line with previous studies suggesting that people from northern European countries tend to be less lonely than their peers in southern and central European countries [24, 54]. Financial and socioeconomic aspects [24], as well as the different characteristics of the health and welfare systems [23] could explain the observed differences across countries. The small differences found in the scores in loneliness across countries and between age groups were similar to those found in previous studies such as the Survey of Health, Ageing and Retirement in Europe (SHARE) [24, 54].

The frequency of contact with members of the network was the only component of the social network having a significant association with health. Fernández-Ballesteros [9] also found a significant correlation between frequency of contact and health in a Spanish sample of people over 65 years. Small differences across countries were found in the relationship between frequency of contact and health, with the association being slightly stronger in Poland than in Finland and Spain. Litwin and Stoeckel [26] also found weak or inconsistent effects of social networks on health outcomes in two different countries.

The results for the present study obtained in all three countries suggest that the subjective perception of loneliness has a strong association with health status, above and beyond what could be explained by covariates such as age, gender, marital status, household size and years of education. Even though this relationship was slightly different across the three countries, previous studies also found differences across countries [54]. The relationship between loneliness and health was likewise reported in studies that also used the UCLA Loneliness Scale [13–15]. Hawkley and Cacioppo [55] proposed a model that explained how loneliness has physical and mental consequences. According to this model, some effects of loneliness are: impairments in attention, cognition, affect, and behavior that activate genetic, neural and hormonal mechanisms, and modifications in the immune functioning, all of which contribute to adverse health outcomes (morbidity and mortality).

The association of subjective feelings of loneliness with health was clearly stronger than the association of the different components of the social network. These results are consistent with previous evidence suggesting that loneliness contributes more strongly to health than any aspect of the social network [56]. Cacioppo and Cacioppo [6] confirmed that loneliness was associated with health problems and the effects of these problems can contribute to early mortality. Steptoe et al. [42] found that loneliness was associated with more health conditions than social isolation, but when they looked at the association with mortality, social isolation had a stronger impact on mortality than loneliness after controlling for several health indicators. As the authors suggest, the fact that loneliness did not have an impact on mortality after controlling for health indicators could be explained by the strong association between loneliness and baseline health.

In the present study, we found a stronger relationship between loneliness and health in the younger population than in older people. It was also found that the mean score for loneliness was higher in the oldest old. This is similar with the results of a previous study by Pinquart and Sörensen [57]. Small differences by gender in the loneliness mean scores were found, as reported in previous studies [58].

To our knowledge, the present study is the first to be carried out with nationally representative samples of several countries using identical methods that disentangled the association of health with different components of the social network and loneliness. Nonetheless, these results must be interpreted with caution and a number of limitations should be borne in mind. Due to its cross-sectional design, the present study is limited by temporality and causality, i.e., it was carried out at a single point in time and gives no indication of the sequence of events—whether an increase in the loneliness level occurred before, during or after the deterioration of the individual's health state. By means of the regression models employed in this study, the dependence of health status on loneliness and social network was described. According to Sokal and Rohlf [59] some evidence regarding the possible causation of changes in health status by changes in loneliness could be obtained. However, this evidence can turn out to be weak, because due to the design of the study, it cannot determine the cause, effect and directionality of the relationships. Moreover, a deterioration of health status can increase the level of loneliness, with changes in loneliness being affected by changes in health status or by the presence of depression. In the work of Peerenboom et al. [60], it was found that depression (as the independent variable) was associated with loneliness (as a dependent variable) even after controlling for confounding factors. Similarly, a recent review of cross-sectional and longitudinal studies focused on loneliness found that loneliness and depressive symptomatology could act synergistically, since these variables have reciprocal influences over time [61]. In addition, the health measure is based on self-reporting. An inherent limitation to the analyses conducted in the present study was that the participants who did not answer the question about their social network or their perception of loneliness were excluded. If a participant was cognitively impaired and not able to respond to the interview, a proxy was asked some questions, but the proxy did not respond to the questions about social networks or loneliness. For this reason, these participants were not included in the present analyses. A socio-demographic comparison was carried out between the included and the excluded samples, and in general terms the differences found had a small associated effect size, indicating that they could be due to the large sample size considered in this study; only high differences in age were found between the included and the excluded sample.

Regarding the response rates, some differences were found across countries. In general terms, even though there are no strict standards, the response rates found in the present study can be considered adequate [62] and similar to the ones found in other general population studies recently conducted in Europe, such as SHARE (global response rate for the ten countries of 61.8%, ranging from 37.6% in Switzerland to 73.6% in France) [63], ELSA (individual response rate of 67%) [64] and TILDA (household response rate of 62%) [65]. The participation was lower in Finland than in the other two countries. This is consistent with a global decrease in response rate there which has been observed in many international epidemiological studies [66]. In the NordChild 2011 survey, the response rate in Finland was 48.06%, a similar rate to those found in the same study in other Nordic countries like Iceland (47.5%), Norway (49.4%), and Sweden (45.7%) [67].

The instruments used to measure loneliness and social networks in the present study have been employed in previous studies and have shown adequate reliability. Although the UCLA Loneliness Scale has only three items, it appears to measure overall loneliness quite well [33]. The scale can be completed in just a few minutes and is adequate for large population health surveys like COURAGE in Europe. It has also been used in previous studies, such as the English Longitudinal Study on Ageing (ELSA) [41, 68]. Regarding the measure of the social networks, previous studies also used the question about the number of contacts in the network [69]. Moreover, the Elderly in Linköping Screening Assessment, carried out by Vikström et al. [70] in Sweden, measured closeness with relatives, friends and neighbors as part of the social network. Moreover, the question regarding frequency of contact with the members of the social network has been used in national surveys in Spain to assess how often old people have face-to-face contacts, talk by phone, or exchange letters/e-mails with the members of their network [71]. However, it is difficult to compare each component of the social network with previous studies, since they report a total score for the social network, and to our knowledge, ours is the first study that disentangled each component to analyze them individually.

As far as we know, this is also the first study that assesses the differential association of health with the components of the social network and the subjective perception of loneliness in representative samples from three countries that represent different European regions [31] with different socio-economic and health characteristics and welfare systems [23]. This investigation is consistent with the previous literature, which shows the importance in public health of loneliness and the components of the social network. It could be relevant to consider the evaluation and screening of feelings of loneliness, and not only the social network, in persons with health problems. Further longitudinal studies are needed in order to be able to infer causality from the associations found in the present study, and to examine the pathways linking loneliness and social networks to health.

## Disclosure

The views expressed in this paper are those of the authors, and do not necessarily represent the views or policies of the World Health Organization.

## Supporting Information

S1 Appendix Questionnaire used in the present study. (DTA)
